# CD25^+^ T-lymphocytes induce CD11b on eosinophils in allergic nasal mucosa

**DOI:** 10.1155/S0962935195000251

**Published:** 1995-03

**Authors:** S. Horiguchi, K. Hisamatsu, K. Tasaka

**Affiliations:** 1 Department of Otorhinolaryngology Yamanashi Medical University 1110 Shimokato Tamaho-cho, Nakakoma-gun, Yamanashi 409-38 Japan; 2 Department of Parasitology & Immunology Yamanashi Medical University 1110 Shimokato Tamaho-cho, Nakakoma-gun, Yamanashi 409-38 Japan

## Abstract

In the allergic mucosa, there is a significant increase in numbers of CD25^+^ cells and activated eosinophils. To determine whether a link exists between the activated T-lymphocytes and tissue eosinophils in nasal allergy, we studied CD25^+^ cells in the nasal mucosa and compared the levels of soluble IL-2 receptor (sIL-2R) both in the serum and the nasal secretions, and further investigated expression of CD11b on eosinophils in the nasal lavage fluids and peripheral blood of patients with nasal allergy. We also examined the effects of the culture supernatant of Con A- and IL-2-activated T-lymphocytes on CD11b expression on eosinophils in the present study. The concentration of sIL-2R in the nasal secretions from patients with Japanese cedar pollinosis (JCP) was significantly higher than that from normal subjects (p < 0.01). The sIL-2R level was significantly higher in the nasal secretions than in the sera in patients (p < 0.01), and CD11b expression on eosinophils from nasal hvage fluid was significandy higher than that of eosinophils from peripheral blood of the same individuals (p < 0.01). The activated T-lymphocytes promoted eosinophil activation with upregulation of CD11b *in vitro*, and eosinophils in the nasal secretions from patients significantly expressed more CD11b *in vivo*. These results indicate that activation of T-lymphocytes is linked to eosinophil activation in nasal allergy.


Mediators of Inflammation 4, 133–137 (1995)

### Inhibition of neutrophil migration by a selective inhibitor of matrix metalloproteinase: analysis by intravital microscopy

**T. Oda,[1*] M. Katori,[1,CA] K. Hatanaka[1] and Y. Nagai[2]**

[1]Department of Pharmacology, Kitasato University School of Medicine, Sagamihara, Kanagawa 228, Japan; [2]Department of Tissue Physiology, Medical Research Institute, Tokyo Medical and Dental University, Tokyo 101, Japan
*Present address: Department of Biology, Faculty of General Education, Yamagata University, Yamagata 990, Japan

[CA] Corresponding Author

**Observation of the microcirculation of the hamster cheek pouch by intravital microscopy revealed five steps of neutrophil migration from the venules after topical application of leukotriene $B_4$ to the microvasculature: rolling along the venular wall (Step 1), adhesion to it (Step 2), disappearance from the vascular lumen (Step 3), presence between the endothelial cells and the subendothelial basement membrane (Step 4) and passage through the basement membrane (Step 5). The present study was performed to examine whether a metalloproteinase inhibitor inhibits neutrophil migration at any of the above five steps. Chymostatin and leupeptin did not inhibit any of these five steps. In contrast, FN-439, a selective inhibitor of matrix metalloproteinase, reduced the number of neutrophils in the perivascular space without affecting Steps 1 to 3. It was concluded that neutrophils may use metalloproteinase (collagenase/gelatinase) to penetrate the subendothelial basement membrane.**

**Key words:** Inhibitor, Metalloproteinase, Microscopy, Neutrophil migration

### Introduction

Migration of polymorphonuclear leukocytes, mainly neutrophils, from the circulation to the extravascular space is one of the cardinal signs of the acute inflammatory response. A study of chemoattractant-induced migration of neutrophils in the microcirculation of the hamster cheek pouch by intravital microscopy[1] has demonstrated five steps from the circulation to the extravascular space. Once chemoattractants such as leukotriene (LT) $B_4$ or fMLP (*N*-formyl-methionyl-leucyl-phenylalanine) are applied over the microvasculature, neutrophil numbers rolling on the inner surface of the venules did not always increase, compared with that before chemoattractants (Step 1), but neutrophils adhere to the endothelial cells (Step 2), pass through the endothelial cell layer (Step 3), remain in the space between the endothelial cells and the subendothelial basement membrane for longer than 30 min (Step 4), as revealed by electron micrography, and finally penetrate the basement membrane of endothelial cells and pericytes (Step 5). A glucocorticosteroid, dexamethasone, inhibits neutrophil migration by selective inhibition of the last step of the penetration of the basement membrane (Step 5).[2]

The aim of the present study was to test whether neutrophils require matrix metalloproteinase in the process of migration from the venules or passage through the basement membrane. The results indicated that Step 5 was inhibited by a selective inhibitor of matrix metalloproteinase (collagenase/gelatinase), suggesting that the passage might be completed if, for example, type IV collagen were cleaved by collagenase/gelatinase.

### Materials and Methods

*Preparation of hamster cheek pouch:* Hamster cheek pouches were prepared as described previously.[1-3] Male golden hamsters (120–150 g, 10–16 weeks old, Nihon SLC, Shizuoka, Japan) were anaesthetized with pentobarbital (Nembutal, Abbot Lab., Chicago, IL, USA) (60 mg/kg body weight, intraperitoneally), and their cheek pouches were everted, cut longitudinally, and extended. The connective tissue was elaborately dissected away to expose the microvasculature of the mucous layer. The thin mucous membrane tissue was spread out in a plastic chamber and superfused with 5 ml/min of warmed Tyrode's solution, which was kept at 37°C. Body temperature was maintained at 37°C by a warming-pad controller positioned directly beneath the hamster. Tracheal intubation ensured spontaneous respiration.

*Microscopic observation and recording of leukocyte behaviour:* The microvasculature of the hamster

© 1995 Rapid Communications of Oxford Ltd cheek pouch was observed under a transillumination microscope (BHA, Olympus Optical Co., Ltd, Tokyo, Japan), using a W40 water immersion lens (×40, Nikon, Tokyo, Japan) and ×10 eyepieces (Olympus Optical Co., Ltd, Tokyo, Japan). Images of the microcirculation were projected onto a colour television (TV) monitor screen (CMM20-7HR, Shibasoku, Tokyo, Japan) via a colour TV camera (CTC-2600, Ikegami Tsushinki, Tokyo, Japan) mounted at the top of the microscope, as described previously.[1-3]

The behaviour of the leukocytes in each experiment were usually recorded with a videotape recorder (VO-5850, Sony Co., Tokyo, Japan) and reviewed after the experiments. The images of individual leukocytes during migration were viewed on the screen. The experimental procedures were performed only once in each animal. The behaviour of a leukocyte over a 100 μm length of a postcapillary venule with a diameter of 13–15 μm was observed on the monitor screen. Over a 30 min equilibration period, leukocytes were rolling along the endothelium of the venules at a lower velocity than were erythrocytes.

*Chemotactic agent and proteinase inhibitors:* Leukotriene (LT) $B_4$ (Paesel, GMBH, Frankfurt, Germany) was used as a chemoattractant. A stock solution of $LTB_4$ (30 μM in absolute ethanol) was kept at −80°C and diluted to 300 nM with Tyrode's solution immediately before use. The superfusion of the cheek pouch with Tyrode's solution was stopped during the experiments. $LTB_4$ (15 pmol/50 μl) was applied to the microvasculature at the observation site with a 50 μl micropipette.

FN-439 is a tetrapeptidyl hydroxamic acid with the structure *p*-$NH_2$-Bz-Gly-Pro-D-Leu-D-Ala-NHOH, which is a selective inhibitor of matrix metalloproteinase (collagenase/gelatinase; Fuji Chemical Industry, Toyama, Japan).[4] A sample of FN-439 was dissolved in Tyrode's solution at a concentration of 1 mM, and 50 nmol/50 μl of the resulting solution was applied to the microvasculature 5 min after a single $LTB_4$ application and every 5 min thereafter for a total of ten times. Chymostatin (an inhibitor of cathepsins A, B and D; the Peptide Institute, Minoh, Osaka, Japan) and leupeptin (an inhibitor of trypsin and cathepsin B; the Peptide Institute, Minoh, Osaka) were separately dissolved in Tyrode's solution, and 50 μl samples of these solutions were applied to the microvasculature in the same way as FN-439.

### Results

Figure 1 shows the effects of the inhibitors of different enzymes on the cumulative number of newly adhering neutrophils over a 100 μm length of a venule during a 50-min period after topical application of $LTB_4$ (15 pmol/50 μl). The total number of newly adhering neutrophils was 32.0 ± 6.0 during this period. Fifty μl of the solutions of the collagenase/gelatinase inhibitor (FN-439) (1 mM), chymostatin (1 mM) and leupeptin (1 mM), applied 5 min after $LTB_4$ and every 5 min thereafter, did not provide any significant inhibitory effect on the cumulative number of neutrophils newly adhering to the venular wall.

FIG. 1. Effects of the proteinase inhibitors on the cumulative number of neutrophils newly adhering to the inner venular wall for 50 min after application of $LTB_4$. $LTB_4$ (300 nM, 50 μl) was applied topically over the microvasculature. Col.I., collagenase/gelatinase inhibitor (FN-439, 1 mM). Chy, chymostatin (1 mM). Leu, leupeptin. These proteinase inhibitors (50 μl) were applied over the microvasculature 5 min before and every 5 min after $LTB_4$ application. The values represent mean ± S.E.M. The numbers at the top of the columns indicate the numbers of animals used.

The numbers of neutrophils that disappeared from the venular lumen as percentages of the numbers of neutrophils that had adhered to the inner wall of venules are shown in Fig. 2. $LTB_4$ induced the disappearance of nearly 100% of the neutrophils, that had adhered to the venular wall, from the vascular lumen through the endothelial layer. None of the inhibitors applied topically inhibited this passage of the neutrophils into the venular wall.

Figure 3 shows the numbers of neutrophils that appeared in the perivascular space, observed up to 90 min after topical application of $LTB_4$. The number of neutrophils increased with time from 30 min after the application. Chymostatin and leupeptin did not significantly modify the numbers of neutrophils in the perivascular space. FN-439, however, significantly reduced these numbers, from 3.0 ± 0.4 to 0.4 ± 0.2 in 30 min, from 12.6 ± 0.8 to 2.2 ± 0.8 in 60 min, and from 19.8 ± 1.0 to 4.4 ± 1.0 in 90 min.

FIG. 2. Effects of proteinase inhibitors on the number of neutrophils that disappeared from the venular lumen as a percentage of the adhering neutrophil count. EC, endothelial cells. For other abbreviations, see Fig. 1. Application of the inhibitors was made in the same way as for Fig.1. The values represent mean ± S.E.M. The numbers of animals were the same as in Fig. 1.

### Discussion

Migration of neutrophils at the level of microcirculation in hamster cheek pouch is not a simple phenomenon, but involves five steps, as reported in the previous paper.[1] Step 1, rolling on the venular wall, may be attributed to the selectin type of adhesion molecule.[5,6] Step 2, adhesion of neutrophils to the venular wall, is initiated by a change in the neutrophil membrane[3] and may be due to activation of the adhesion molecule, CD11/CD18, since the adhesion of neutrophils was inhibited by the antibody against rat CD18.[7] The observation in the present experiments that the number of neutrophils adhering to the venules was not reduced by chymostatin, leupeptin or FN-439 may indicate that in this adhesion step, neutrophils did not require proteolytic enzymes such as cathepsins A, B and D or metalloproteinases for adhesion to the endothelial cells. Involvement of other proteinases in this process cannot be excluded in the present study.

These enzyme inhibitors also did not suppress the rate of disappearance of neutrophils from the venular lumen (Step 3), indicating that the passage of the neutrophils between the endothelial cells may not require the action of proteolytic enzymes.

We were unable to study the effect of inhibitors on the neutrophils which remained in the venular wall between the endothelial cell layer and the venular basement membrane (Step 4), owing to the lack of

FIG. 3. Effects of proteinase inhibitors on the neutrophil counts in the perivascular space 30, 60 and 90 min after application of $LTB_4$. For the abbreviations, see Fig. 1. Application of the inhibitors was made in the same way as for Fig. 1 up to 90 min. The values were compared with that for $LTB_4$ alone. The counts after application of a collagenase/gelatinase inhibitor (FN-439) were significantly reduced (*$p < 0.05$). The numbers of animals were the same as in Fig. 1.

basement membrane (Step 4), owing to the lack of a method for evaluation. Nevertheless, the fact that the number of neutrophils counted in the perivascular space was not affected by topical application of chymostatin or leupeptin indicated that these proteinase inhibitors did not affect the movement within the venular wall. Alternatively, it suggested that proteolytic enzymes such as cathepsins A, B and D were not required for neutrophils to transmigrate through the basement membrane to the perivascular space (Step 5). In contrast, the number of neutrophils counted in the perivascular space after topical application of $LTB_4$ was markedly reduced by FN-439. FN-439 was reported[8] to inhibit the human skin collagenases, human granulocyte collagenase and tadpole collagenase at $IC_{50}$ values of 1.3 μM, 0.9 μM and 1.1 μM, respectively and granulocyte gelatinase and skin fibroblast stromelysin at $IC_{50}$ values of 30 μM and 150 μM, but no apparent inhibition was observed against thermolysin, urease, trypsin, α-chymotrypsin, plasmin and human granulocyte elastase. Alkali-induced corneal ulceration in rabbit eyes was prevented by FN-439.[9] The inhibition of neutrophil migration to the perivascular space observed in the present study clearly indicated that the neutrophils use matrix metalloproteinase to cleave a part of the basement membrane or type IV collagen for passage through this membrane. The videotape image in the present study revealed that neutrophils were going out, one by one, from a part of the venular wall to the perivascular space 1 h after the application of $LTB_4$.

In vessel wall constructs *in vitro*,[10] using endothelial cells from human umbilical vein, which were cultured on type I collagen and synthesized continuous subendothelial basement membrane, the permeability increase of Monastral Blue B colloid by neutrophils and neutrophil emigration were not prevented by inhibitors of the serine protease, elastase and cathepsin G (DFP, aprotinin, leupeptin, pepstatin and others), by a natural metalloproteinase inhibitor (a tissue inhibitor of metalloproteinases, TIMP) and by inhibitors of endoglycosidase and heparanase (heparin). These *in vitro* results, showing that the loss of basement membrane integrity associated with neutrophil diapedesis was independent of the neutrophil proteases, were consistent with those in the present study, but the failure of the inhibition by TIMP might be due to rapid destruction of the endogenous inhibitor, whereas FN-439 was reported to be resistant to hydrolysis by pronase.[8]

It has been reported[11] (1) that human neutrophils secrete specific granule contents (e.g., lysozyme and $B_{12}$-binding protein), but not azurophil granule contents (e.g., β-glucuronidase), during migration across the micropore filters of modified Boyden chambers in response to a chemoattractant; (2) that specific granule products, but not azurophil granule products, were detected in the extracellular fluid of experimental human skin chambers or skin blisters *in vivo*, coincident with the influx of exudate neutrophils, and (3) that a loss of specific granules, but not azurophil granules, from exudate cells was demonstrated in these models. These findings may well account for the results in the present studies, since collagenase is reported to be present in specific granules, while elastase is found in the azurophil granules of human neutrophils.[12]

It is not clear whether metalloproteinases are synthesized in neutrophils during their presence within the venular wall for longer than 30 min in the hamster cheek pouch.[1] It was reported[13] that adhesion to covalently immobilized peptides containing the Arg-Gly-Asp sequence that were derived from fibronectin induced collagenase and stromelysin gene expression. In an *in vitro* model of a barrier of endothelial cells,[14] neutrophils, placed in the upper compartment of a chemotaxis chamber, extend their pseudopods into filters with a pore size of less than 0.6 μm, in response to the gradient of the chemoattractant. The bulk of the cytoplasmic granules, the centriole and the Golgi apparatus are toward the front of the cell just behind the hyaline- and microfilament-filled pseudopods. This location may facilitate the secretion of granular contents towards the front portion of the cell during migration. Pretreatment of rabbits with cycloheximide or actinomycin D was reported to inhibit neutrophil migration induced by interleukin-1, tumour necrosis factor-α or endotoxin, but not that by $C_{5a}$-des-Arg, fMLP or $LTB_4$.[15] In contrast, 72-kDA gelatinase was induced on T cells, when they bind to VCAM-1 on the surface of endothelial cells.[16]

We reported previously[2] that dexamethasone inhibits migration at Step 5. This is in accord with the finding that induction of the gene expression of metalloproteinase in fibroblasts by the Arg-Gly-Asp sequence was inhibited by dexamethasone.[13] It cannot be concluded whether metalloproteinases are synthesized in neutrophils or the proteinases already synthesized are secreted, but these results, taken together, suggest that the period in which neutrophils remained in the space between the endothelial cells and the subendothelial basement membrane in the present experiment, may be necessary for the neutrophils to synthesize the metalloproteinase or to prepare to secrete this enzyme.

Our present results suggest that a metalloproteinase inhibitor such as FN-439 may be a potential therapeutic drug that would prevent neutrophil migration in inflammatory diseases such as rheumatoid arthritis. In conclusion, neutrophils may use metalloproteinase or collagenase/gelatinase, at the stage of passage through the subendothelial basement membrane during their transmigration.

### References

1. Oda T, Katori M, Hatanaka KS. Five steps in leukocyte extravasation in the microcirculation by chemoattractants. *Mediators of Inflammation* 1992; **1**: 403–409.
2. Oda T, Katori M. Inhibition site of dexamethasone on extravasation of polymorphonuclear leukocytes in the hamster cheek pouch microcirculation. *J Leukocyte Biol* 1992; **52**: 337–342.
3. Nagai K, Katori M. Possible changes in the leukocyte adhesion to the venular walls induced by leukotriene B4 and fMLP in the microvasculature of the hamster cheek pouch. *Int J Microc: Clin Exp* 1988; **7**: 305–314.
4. Odake S, Okayama T, Obata M, Moriwaki T, Hattori S, Hori H, Nagai Y. Vertebrate collagenase inhibitor. II. Tetrapeptidyl hydroxamic acids. *Chem Pharm Bull* 1991; **39**: 1489–1494.
5. Abbassi O, Lane CL, Krater S, *et al.* Canine neutrophil margination mediated by lectin adhesion molecules-1 *in vitro*. *J Immunol* 1991; **147**: 2107–2115.
6. Kishimoto TK, Warnock RA, Jutila MA, *et al.* Antibodies against human neutrophil LECAM-1 (LAM-1/Leu-8/DREG-56 antigen) and endothelial cell ELAM-1 inhibit a common CD18-independent adhesion pathway *in vitro*. *Blood* 1991; **78**: 805–811.
7. Hatanaka K, Katori M. Detection of polymorphonuclear leukocytes adhered on venular endothelial cells. In: Tsuchiya M, Asano M, Ohshima N, eds. *Microcirculation Annual 1992*. Tokyo: Nihon-Igakukan, 1992; 129–130.
8. Odake S, Morita Y, Morikawa T, Morikawa T, Yoshida N, Hori H, Nagai Y. Inhibition of matrix metalloproteinases by peptidyl hydroxamic acids. *Biochem Biophys Res Comm* 1994; **199**: 1442–1446.
9. Kigasawa K, Murata H, Morita Y, *et al.* Inhibition of corneal ulceration by tetrapeptidyl hydrozamic acid. *Jpn J Ophthalmol* 1995; **39**: 35–42.
10. Huber AR, Weiss, SJ. Disruption of the subendothelial basement membrane during neutrophil diapedesis in an *in vitro* construct of a blood vessel wall. *J Clin Invest* 1989; **83**: 1122–1136.
11. Wright DG, Gallin JI. Secretory responses of human neutrophils: exocytosis of specific (secondary) granules by human neutrophils during adherence *in vitro* and during exudation *in vivo*. *J Immunol* 1979; **123**: 285–294.
12. Bainton DF. Phagocytic cells: developmental biology of neutrophils and eosinophils. In: Gallin JI, Goldstein IM, Snyderman R, eds. *Inflammation. Basic principles and clinical correlates*. New York: Raven Press, 1988; 265–280.
13. Werb Z, Tremble PM, Behrendtsen O, Crowley E, Damsky CH. Signal transduction through the fibronectin receptor induced collagenase and stromelysin gene expression. *J Cell Biol* 1989; **109**: 877–899.
14. Malech HL, Root RK, Gallin JI. Structural analysis of human neutrophil migration: centriole, microtubule and microfilament orientation and function during chemotaxis. *J Cell Biol* 1977; **75**: 666–693.
15. Cybulsky MI, McComb DJ, Movat HZ. Protein synthesis dependent and independent mechanisms of neutrophils emigration. *Am J Pathol* 1989; **135**: 227–237.
16. Romanic AM, Madri JA. The induction of 72-kDa gelatinase in T cells upon adhesion to endothelial cells in VCAM-1 dependent. *J Cell Biol* 1994; **125**: 1165–1178.

ACKNOWLEDGEMENTS. We wish to thank Mrs M. Ogino and Mrs C. Shim for their skilful technical assistance. We would like to express sincere thanks to Mr H. Ishikawa and Mr M. Soma for manufacturing mechanical devices. This work was partly supported by Grant-in-Aid for Scientific Research (B)(#02454497).

**Received 25 November 1994; accepted 13 January 1995**
